# Are all researchers male? Gender misattributions in citations

**DOI:** 10.1007/s11192-016-2192-y

**Published:** 2016-12-30

**Authors:** Michał Krawczyk

**Affiliations:** University of Warsaw, Warsaw, Poland

**Keywords:** Academic citations, Gender misattribution, Gender-science stereotype, Gender discrimination

## Abstract

I screen academic literature for cases of misattribution of cited author’s gender. While such mistakes are overall not common, their frequency depends dramatically on the gender of the cited author. Female scholar are cited as if they were male more than ten times more often than the opposite happens, probably revealing that citers are influenced by the gender-science stereotype. The gender of the citing author and the field of study appear to have only limited effect.

## Introduction

Numerous studies confirm that the social role of a scientist is strongly associated with the male gender (Steinke et al. 2007; Cvencek et al. 2011). While this is understandable in view of the dramatic gender disparity in the recorded history of science, the stereotype can prove harmful for the ever-increasing group of female researchers. Indeed, a woman involved in research or even dissemination of science can still cause a surprise. A spectacular example of that could be seen when Elise Andrew, using her newly created Twitter account, revealed herself to be the one behind the popular Facebook blog *I Fucking Love Science (IFLS)* (Holpuch 2013). The effect was perhaps exacerbated by the profanity of the site’s title and the language occasionally used there, which might have been perceived as more appropriate for a man. In any case, thousands of social media users expressed their shock or disbelief, often making comments signaling gender cliches.

If the specific instance of a woman-science combination represents a cognitive dissonance, two general strategies of dealing with it seem possible. First, one can come to believe that this is in fact no good science. For example, in what seems to be an interesting case of hindsight bias, a FB user named Pierre Rodriguez wrote “I had an intuition not to take the posts [of IFLS] seriously…”. Much more importantly, some evidence has been found that women’s academic work is evaluated systematically differently from that of men. For example, Budden et al. (2008) found that the introduction of a double-blind review process by Behavioral Ecology significantly increased the fraction of published papers that were first-authored by a woman. Such conclusions were, however, often questioned, e.g. Engqvist and Frommen (2008) failed to confirm a significant effect when applying a more rigorous statistical procedure. Likewise, in their review of much of this literature Ceci and Williams (2011) concluded there was no field evidence that discrimination against women by journals, job committees and granting bodies continues today. This line of research, however, is impeded by such issues as self-selection and difficulty in comparing research quality. To address these problems, some scholars have turned to controlled experiments, in particular investigating if the evaluation of an academics text depends on the gender fo the author (Borsuk et al. 2009a; Krawczyk and Smyk 2016).

The other way out of the cognitive dissonance involves overlooking or ignoring the fact that the contributor is a woman in the first place. Some social media fans of IFLS commenting on the “newly revealed” identity of Elise Andrew noted that the blog host had in fact given interviews before and otherwise identified herself as a women. Yet, because these hints had been more subtle, generally speaking they had been conveniently overlooked by the audience. Until now, however, there seems to be no systematic evidence on this kind of refusal to recognize the identity of female scientists. This project is sought to fill in this gap.

Towards this end I look for instances, in which academic work was incorrectly cited, in that the gender of the author was misattributed. More specifically, I compare the cases in which a female author would be referred to as “he” to the cases where a male author would be referred to as “she”. If a large, systematic difference in the prevalence of these two types of mistakes is found, it is proposed to be a manifestation of the adoption of the male-science stereotype by the citers. At the same time, such a practice reinforces the biased perception, in particular contributing to the lack of relevant role model perceived by female students and young researchers (Bettinger and Long 2005). It may also adversely affect individual scholars, whose gender is misattributed, for it probably reduces their recognizability.

In order to identify cases of gender misattribution, 2893 often-cited single-authored (by a man or a women) academic publications from all fields of study were selected (see supplementary materials for details of the research procedure). Next, papers citing them were screened for cases of misattribution of gender of the cited author.

On the basis of literature reviewed before I hypothesized that female scholars would have their gender misattributed more often than males and that the effect will be will be highest in older publications. Further interesting interesting research questions concern the gender of the citer. We may speculate that males and researchers in male-dominated fields may misattribute females’ gender most often.

## Relevant scientometric literature

The justification for this research in based on the following premises. First, being cited plays a tremendous role in developing an academic career, see e.g. the works listed in Bornmann and Daniel (2008). Second, giving credit to relevant sources is not the only motivation nurtured by researchers; in particular, social networks play a role: authors cite primarily works by authors with whom they are personally acquainted (White 2001). There is some evidence that gender stereotypes may affect such choices. For example Davenport and Snyder (1995) did observe that women are cited less in the sociology journals they consider. This is not true of all studies, however, for example Borsuk et al. (2009b), Ledin et al. (2007) found no such effect and Powell et al. (2009) reported the opposite. Overall, difference in the overall number of citations (e.g. measured by* h*-index) that a representative male vs. female scholar acquires Geraci et al. (2015) is generally driven by the larger number of citable papers men typically produce rather than mean per-publication citations; moreover, it may have disappeared in the youngest generation of scholars Arensbergen et al. (2012). Again, even if differences in per-publication citation patterns were found, it cannot be immediately taken as evidence for discrimination, as controlling for quality of publications and base rates is notoriously difficult.

Third, studies such as Broadus (1983), Eichorn and Yankauer (1987) show that references in academic literature are surprisingly often incorrect, with estimates ranging between 10 and 60% (although of course most mistakes are minor). As Evans et al. (1990) put it, “The data support the hypothesis that authors do not check their references or may not even read them” (p. 1353). As a result, it is common that credit is not given to the original source of the idea, e.g. because a review paper is cited instead (Teixeira et al. 2013). Altogether, it appears plausible that authors’ genders do get misattributed sometimes, that it happens more often to female scholars and that it has some impact on their careers and the perception of the role of women in science in general.

## Results

Table 1 presents prevalence of misattributions by cited author’s gender and field of study.

**Table 1:** Number of papers, whose authors’ gender was misattributed at least once, by gender and broad field of study

Broad field	Author	
	Male	Female
Bio/med	0	1
	(283)	(133)
Biz, econ	1	15
	(340)	(197)
Phys/chem/engi	0	7
	(652)	(102)
Social sc, arts, hum	2	30
	(478)	(708)

The total number of citable papers checked is given in the parentheses. For example, just one out of 133 highly-cited female-authored papers in biomedical research has been subject to gender misattribution in at least one citing paper

Three main findings should be noted. First, the prevalence of gender misattributions is quite low. Overall, of the 2893 sources checked, authors of but 57 (1.97%) have been subject to gender misattribution (there were 66 mistakes in total, because some papers have been incorrectly cited more than once). Second, a striking effect of gender can be easily identified: I only found four male-turned-female mistakes (concerning three papers). For females, as many as 53 papers (4.65%) are at least once miscited. Given that there are ca. 4 gender attributions per source paper in our sample, 1.16% of all attributions of single female authors turn out to be incorrect, whereas the rate for males is .04%. Third, it appears that there is an effect of the field of study—female-turned-male mistakes are most common in biz and econ, then social sciences, arts and humanities and essentially never happen in bio/med. This effect cannot be explained in terms no gender being attributed at all to authors of of bio/med publications. For example, the number of total correct attributions in bio/med is quite comparable to that of biz and econ (2.57 vs. 2.92). To validate that the differences are significant, a simple probit regression was run, see Table 2.^1^

**Table 2:** Impact of field of study, publication year and number of citations on probability of misattribution—probit analysis

Mistake	Coef.	SE	*z*	$P>|z|$	(95% CI)	
Broad field						
Biz, econ	0.905	0.387	2.34	0.019	0.147	1.663
Phys./chem./engi.	0.867	0.410	2.11	0.035	0.063	1.671
Social sc., arts, hum.	0.636	0.372	1.71	0.087	−0.0920	1.365
Citations	0.0011	0.0003	3.57	0.000	0.0005	0.002
Citations squared	−1.99e−07	9.43e−08	−2.10	0.035	−3.83e−07	−1.37e−08
Year	0.004	0.005	0.69	0.489	−0.007	0.014
Cons	−10.427	11.167	−0.93	0.350	−32.314	11.460
*N*	1139					

Compared to the base category of bio/med, mistakes are more common in biz/econ as well as in physics, chemistry or engineering and only weakly more common in social sciences, arts and humanities. While establishing the fraction of female authors in such fields is quite difficult, one can probably safely bet that it highest in the (intermediate) category of arts and humanities; there thus seems to be no straightforward relationship between visibility of females in the field and prevalence of mistakes. Note also that, contrary to the hypothesis, there is no effect of the year in which the source was published. As evidenced in the Supplementary Materials, the gender of the citing author did not seem to matter.

## Summary and conclusions

Overall, few gender misattributions are made. This is probably mostly because there is rarely a need to make an attribution in the first place, so presumably authors who choose to do so tend to be those, who are quite confident they do it correctly. Indeed, many of them may simply know the cited author personally. Moreover, research assistants, referees, editors and proofreaders likely successfully deal with most remaining problems. In this sense, our estimates that more than 1 in 100 gender-specific citations of women are incorrect (compared to essentially zero for male authors) do not seem that low.

The plausible explanation is that the gender-science cliche remains strong in (some) authors, so that they do not feel the need to check. Hopefully, with more females reaching high academic positions and publishing successfully, also in traditionally male-dominated fields, the tendency will come to an end. For instance, the Fields Medal being awarded to a woman, Maryam Mirzakhani, for the first time in 2014, may help prove beliefs that “girls can’t do math” wrong. By contrast, individual female researchers’ understandable unwillingness to emphasize their gender, may slow down the process.

On the bright side, as the prevalence is low, these mistakes per se probably do not significantly contribute to lower awareness of female researchers’ achievements.

## Footnotes

1 Again, the independent variable is a dummy indicating whether there is at least one misattribution for given source paper or not. In other words, I do not distinguish here between papers whose authors’ gender has been misattributted once and those for which it happened more than once (which makes no difference anyway because there are just six papers in the last group). This regression only includes female-turned-male mistakes.
